# Characterizing the level of urban Flood vulnerability using the social-ecological-technological systems framework, the case of Adama city, Ethiopia

**DOI:** 10.1016/j.heliyon.2023.e20723

**Published:** 2023-10-06

**Authors:** Bikila Merga Leta, Dagnachew Adugna

**Affiliations:** a Ethiopian Institute of Architecture, Building Construction & City Development, Addis Ababa University, Addis Ababa, Ethiopia; b Addis Ababa Science and Technology University, Addis Ababa, Ethiopia

**Keywords:** Characterization, Social-ecological-technological systems, GIS, MCDM-AHP

## Abstract

This study characterizes the flood vulnerability of Adama City, Ethiopia, where the city faces high flood vulnerability due to its unplanned urbanization in low-lying floodplain areas surrounding deforested mountains and ridges. The study applied an interlinked Social-Ecological-Technological-Systems (SETS) vulnerability framework using a GIS-based Multi-Criteria Decision-Making and Analytical Hierarchy Process (MCDM-AHP). The framework analyzed exposure, sensitivity, and adaptive capacity to flooding for each of the three SETS domains. The study analyzed 18 variables at the city level within each SETS domain. The result revealed that clusters of flood-vulnerable areas were identified by each SETS domain showing the concentration of flood vulnerability in the study area and the need to consider prompt adaptive mechanisms to severe and recurring flooding. The finding has significant implications for holistic approaches to sustainable cities. Moreover, the reduction of complex urban flood vulnerabilities according to their priority as individual or combined solutions for decision-makers and professionals in early warning and flood management systems is the other contribution of the study.

## 1 Introduction

Flooding has become a significant global hazard that has affected the lives of millions of people. Recent studies have revealed that approximately 1.81 billion people, accounting for 23 % of the global population, are exposed to considerable flood risk [1,2]. As a result of climate change, floods have become one of the most frequent natural disasters and are expected to occur more frequently in the future [[3], [4], [5]]. The consequences of floods extend beyond physical damage, posing a substantial threat to human health, well-being, and the economy, as well as causing harm to ecosystems [1,6,7]. In terms of economic losses alone, floods cause over $40 billion in damages annually across the globe [8]. Furthermore, it is projected that by 2050, an additional two billion people will be at risk of flood disasters [9]. Urban areas situated in floodplains are particularly vulnerable to floods and face heightened risks due to the amplification of flood magnitudes [1,10].

Flooding in cities has been widely attributed to urbanization in floodplain areas [[11], [12], [13]]. Urbanization increases population and building densities, which directly increases surface sealing leading to rapid amplification in the volume and velocity of surface runoff [14,15]. Urbanization in floodplain areas increases the risk of flooding due to increased peak discharge and volume and decreased time to peak [[16], [17], [18]]. Urban watersheds, on average, lose 90 % of the storm rainfall to runoff, whereas non-urban forested watersheds retain 25 % of the rainfall [19]. The floodplain area is highly vulnerable to flooding due to human activities such as the presence of structures, lack of regulations for their maintenance, and deforestation [20,21].

Vulnerability to flooding in urban areas is determined by analyzing the components of exposure, sensitivity, and adaptive capacity. According to Chang, Pallathadka [15], the SETS framework was developed for assessing urban flood vulnerability through 18 indicators representing social, environmental, and technological dimensions. Similarly, Van, Tuan [22] established a flood vulnerability index based on social, economic, environmental, and physical elements. Furthermore, Kashyap and Mahanta [23] proposed an approach combining characteristics associated with exposure, sensitivity, and adaptive capacity [4].

Urban floods are one of the most frequent natural disasters, resulting in severe economic damage and loss of lives [23]. Therefore, tools used to assess urban flood vulnerability are essential. The SET framework is one such tool that incorporates selected exposure, sensitivity, and adaptive capacity indicators in three domains – Social, Ecological, and Technological Systems [4,15]. The social domain includes indicators such as population density, age distribution, and income level; the ecological domain includes indicators such as land use and vegetation cover; and the technological domain includes indicators such as infrastructure quality and availability of emergency services [15]. The SETS framework has been widely utilized in various research studies, encompassing areas such as urban ecosystem services [24], urban flood vulnerability [15], metropolitan changes [25], climate change adaptation [26], and urban sustainability transformations [27]. This framework offers a holistic approach to vulnerability assessment by effectively integrating social, ecological, and technological factors [15]. Additionally, the incorporation of geographic information systems (GIS) enhances the accuracy and precision of the assessment process [4,22,23].

Assessment of flood vulnerability is recognized as a pre-emptive undertaking for hazard management and planning [28]. Researchers widely acknowledged that vulnerability consists of three main components: degree of exposure, sensitivity, and adaptive capacity [29]. Studies have also shown that both physical factors, such as access to infrastructure and services, and socio-economic characteristics, such as population density and income levels, are important indicators for assessing flood risk [4]. Case studies from Santiago de Chile [30] and Côte d'Ivoire [4], utilizing an indicator-based approach proved to be successful in accurately assessing urban flood vulnerability. Furthermore, considering social aspects in addition to technological elements is becoming increasingly important for understanding more complex issues regarding the vulnerability of communities that may not have access to technology or information about floods [15,31]. To build cities that are resilient to extreme weather events and climate change impacts, policymakers and urban planners must understand the drivers of flood vulnerability in their municipalities [4,15,32].

Urban flooding has become a major issue in developing countries, particularly in Africa, due to rapid urbanization and population growth [[33], [34], [35], [36], [37]]. Inadequate land use management practices and climate change effects resulting in natural disasters such as earthquakes, cyclones, landslides, and floods compound this [[38], [39], [40]]. Additionally, increased rooftops, parking lots, and asphalt surfaces contribute to faster floodwater run-off than vegetated lands and bare soils [41]. The impact is worse in these areas due to inadequate housing facilities, infrastructure, water availability, and heightened exposure to pathogens [42,43].

Urban flooding has emerged as a significant concern in Ethiopia due to various factors including inadequate urban drainage infrastructure, insufficient land use planning measures, the absence of early warning systems, and a lack of coordination in flood management activities at both federal and local levels. According to the National Disaster Risk Management Commission [44], Ethiopia is home to twelve major river basins, with Adama City situated in the Awash River Basin. The Awash River Basin has a history of recurrent severe flooding incidents, posing a greater risk to human well-being and property damage compared to other natural disasters.

Recent research highlighted the increasingly significant influence of flood issues in Adama City, given the increasing number of people, economic activities, and ecosystems affected by their unfavorable consequences [[45], [46], [47]]. The historical records demonstrate that floods have had a more detrimental effect on Adama City than any other natural disaster [48]. Though varying in severity with time, urban flooding still represents one of the greatest hazards to Adama City due to its vulnerability to inundation. In addition to having a tangible impact on property, transport, and other critical infrastructure, flooding have far-reaching indirect effects that disrupt communities for extended periods. These include water contamination, fatalities, increased traffic congestion leading to delays in the provision of services, and loss of housing, crops, and livestock [[49], [50], [51]].

Multi-criteria decision-making techniques offer a robust and precise evaluation of urban flood vulnerability, allowing for a comprehensive and accurate assessment. This approach effectively identifies the key factors that significantly contribute to flood risk and determines their relative importance in the decision-making process [[52], [53], [54], [55]]. The efficiency of multi-criteria decision-making in analyzing data and making informed decisions has led to its widespread adoption as a tool for assessing flood vulnerability [[56], [57], [58]]. Previous studies on urban flood vulnerability assessment have primarily relied on multi-criteria decision-making methods, which often fail to explicitly consider the different domains and dimensions of vulnerability. These methods have limitations such as a limited set of criteria and factors [15,28], lack of stakeholder engagement [59], and simplistic weighting and aggregation techniques [60]. Consequently, the assessments provided by these studies are challenging to implement in practical urban flood management.

In line with this, the objective of this study is to characterize the level of urban flood vulnerability in Adama City using SET frameworks. The study builds upon previous work done by Chang, Pallathadka [15], which utilizes the same SET vulnerability framework to evaluate six cities in the US for fluvial/riverine flooding using equal weighting for eighteen indicators. However, this study incorporates stakeholder perspectives to prioritize the indicators within the local context through the utilization of multi-criteria decision-making techniques in pluvial flood vulnerability analysis. This assessment examines various dimensions of vulnerability, including exposure, sensitivity, and adaptive capacity to gain a deeper understanding of the intricate nature of urban flood vulnerability. It is important to note that this study stands out due to its application of the SET framework to assess both separate and combined domains of vulnerability. Moreover, it is assumed that this study is the first to use the SET framework with an Analytical and Hierarchical Process as a whole, and the first to use the SET framework for developing countries in particular. The results will assist city manager and practitioners in improving contextual solutions and promoting sustainable approaches to mitigate the risks associated with flooding in Adama City.

## 2 Methodology

### 2.1 Description of the study area

The present study was conducted in Adama City, Ethiopia. Adama is one of the fastest-growing and second-most populous cities in Ethiopia. It was founded in 1916 on a flat terrain surrounded by plateaus; mountain ranges, and ridged topography, located within the Awash River Watershed and the East African Rift Valley. The study area is located between 8°26′15″– 8° 37′00″ N, and 39° 12′15″- 39°19′45″ E.

The altitude of the study area ranges from 1489 to 1976 m above sea level (m a.s.l), and is located 99 km southeast of Addis Ababa, the capital of Ethiopia with a total area of 30,865.4 ha. The city has two extreme points located at its eastern and northwestern borders. The lowest point is situated on the eastern edge, featuring a flat, low-lying area, which collects floodwater from ‘Borticha’ ridge (Ganda Dabe Solloke), and the eastern edge of ‘Migira’ ridges, forming seasonal ponds. The highest elevation point is found at the northern tip of the Wind Farm II substation, with an altitudinal range measuring up to 487 m. This wide altitudinal variation contributes to the presence of a variety of tropical and subtropical microclimates in the region.

The 2007 census conducted by the Central Statistics Agency of Ethiopia (CSA) revealed that Adama City had a population of 222,035 people and experienced a 5.4 % growth rate, exceeding the average urban growth rate of Ethiopia which was 2.5 %. The Adama City Structure Plan Revision [61], reported that the total population within the administrative boundary of 30,865.4 ha was 431,202 with a doubling rate of 13 years, which is comparatively quicker than the national doubling time of 23 years. This growth has been attributed to its strategic geographic position along the Addis Ababa - Djibouti railway and proximity to Addis Ababa. According to Adama City Structure Plan Revision [61], the rapid population growth in Adama has led to overcrowding near floodplains, resulting in an elevated risk of recurrent floods due to the city's landscape. In addition, a study examining growth rates between 2004 and 2016 indicates that Adama has observed an annual change rate of almost 9 %, putting it on the list of Ethiopia's most rapidly growing cities [62]. The accelerated expansion and surge of the population also puts pressure on infrastructure and public services as well as exposing the local natural environment to a higher risk of degradation [63].

**Fig. 1:**
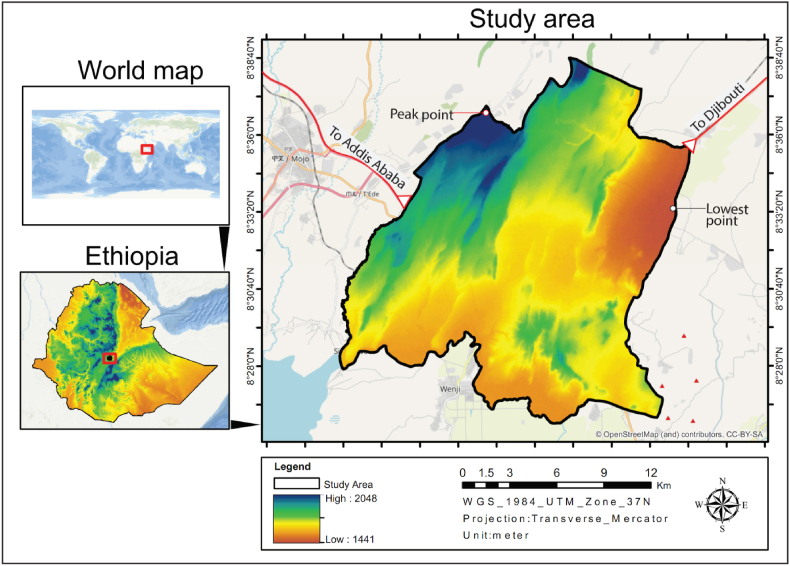
Location map of the study area.

According to National Meteorology Agency [64], Adama City owns a hot semi-arid climate accompanied by an average annual temperature of 20.7 °C. Furthermore, it experiences rain from June through September with an average annual rainfall of 866.25 mm; May is the warmest month at 22.7 °C, and December is the coldest and driest month at 18.3 °C on average. Fig. 1 shows the location map of the study area.

### 2.2 Data sources

The socio-demographic, ecological, and technological frameworks in Adama City were collected both from primary and secondary sources. The secondary data was collected from different bureaus and websites. The spatial data obtained from the Adama city administration was updated from field visits and Google Earth digitalization. For analysis, the collected data was transferred into Arc GIS (version 10.7.1), as presented in Table 1.

**Table 1:** The selected SETS flood vulnerability indicators together with their source.

No	Category	Variables	Abbrevation	Data sources
1	Social exposure	Total population	PT	www.worldpop.org (assessed in March 2023)
2		Population density	PD	www.worldpop.org (assessed in March 2023)
3	Social sensitivity	Percentage of children <5 years	PC	www.worldpop.org (assessed in March 2023)
4		Percentage of population >65 years	PO	www.worldpop.org (assessed in March 2023)
5	Social adaptive capacity	Percentage of women	PW	www.worldpop.org (assessed in March 2023)
6		Percentage of people who are unemployed	PU	ACA 2020
7	Ecological exposure	Slope variation	S	ASTER- Global DEM with 12.5 m size grids.USGS: (http://earthexplorer.usgs.gov/)(accessed in February 2023.
8		The proximity of ecosystem to toxic release sites	PE	Adama City Environmental Protection Bureau, field visit, and google earth digitization
9	Ecological sensitivity	Combination of shape index and average patch size	SP	Developed from ACA vegetation layers, field visit, and google earth digitization
10		Percentage of bare soil within the area	BS	World Land Resource Center, Ethiopia
11	Ecological adaptive capacity	Percentage of wetland within the area	W	Adama City Environmental Protection Bureau, field visit, and google earth digitization
12		Productivity based on Normalized Difference in Vegetation Index (NDVI)	PNDVI	Sentinel-2A Satellite Image with 10 m size grids. Downloaded from USGS (http://earthexplorer.usgs.gov/) acquired in February 2023
13	Technological exposure	Building area (percentage of building area within the area)	BA	ESRI: https://livingatlas.arcgis.com/Landover/.acquired in February 2023
14		Critical infrastructure (CI) (wastewater, polluted industries, gas terminal) in the area	CI	Adama City Environmental Protection Bureau, field visit, google earth digitization
15	Technological sensitivity	Road density	RD	ACA, field visit, google earth digitization
16		Fractional Impervious surface	FIS	Developed from NDVI (Sentinel-2A Satellite Image with 10 m size grids. Downloaded from USGS (http://earthexplorer.usgs.gov/) assessed in February 2023)
17	Technological adaptive capacity	Green Infrastructure (GI) density	GI	ACA, Field visit, google earth digitization
18		Emergency centers (distance of emergency centers (e.g., hospitals, schools, community centers))	EC	ACA, Field visit, google earth digitization

The above SETS variables were developed based on Antwi, Boakye-Danquah [65]; Babanawo, Mattah [66]; Chang, Pallathadka [15]; and Salami, von Meding [29].

### 2.3 Methods

This study utilized the SET frameworks and employed GIS-based AHP to assess the flood vulnerability of Adama City. The analysis encompassed 18 variables, which were categorized into three domains: social, ecological, and technological aspects. These variables were processed and rasterized on a scale of 1–5, representing very low to very high vulnerability levels. The reclassification of the variables was conducted in a GIS environment using the reclassify tool from spatial analysis tools. The resulting data was rescaled and geo-referenced with the same coordinate system of WGS_1984_UTM_Zone_37 N.

To determine the importance of each flood-influencing factor, an AHP model was employed. This involved interviewing 20 experts and 20 local elders to gather their insights on the most significant factors contributing to flooding in the study area. The pair-wise comparison matrix was used to calculate the prioritization of these factors, with each factor's weight expressed as a percentage value between 0 and 100 %. The sum of all weights equaled 100 % to ensure consistency.

The ranking of each reclassified factor was based on a combination of literature review, expert interviews, and residents' input. Factors were ranked on a scale of 1–5, with rank 5 indicating the highest level of influence and rank 1 indicating the lowest. The assigned weight for each factor reflected its contribution to flooding relative to other factors.

**Fig. 2:**
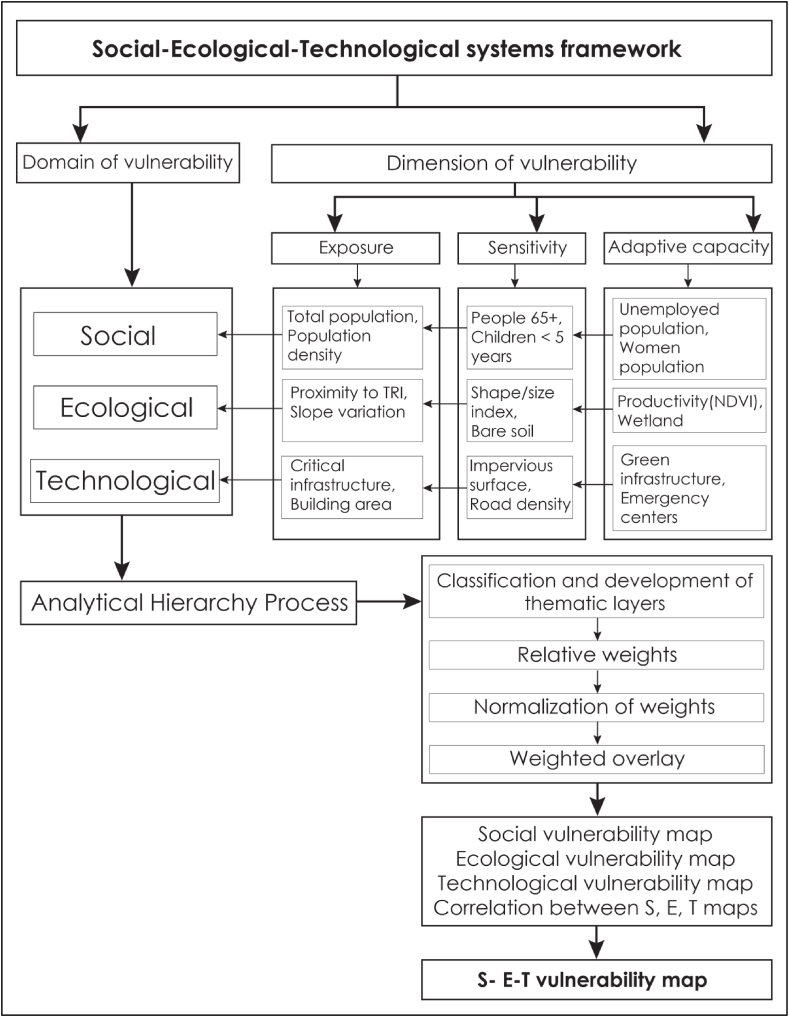
Flowchart showing the domains and dimensions of flood vulnerability of the study area.

Using ArcGIS (version 10.7.1), a multi-criteria analysis was conducted to produce the flooding vulnerability map. This approach allowed for a comprehensive characterization of the various factors contributing to flooding vulnerability in the study area. The resulting map provides valuable insights into the level of flood vulnerability in Adama City. The general workflow followed by this study is represented in Fig. 2.

#### 2.3.1 Methods of mapping SET variables

The spatial population distribution of the 2020 population count of the Ethiopia raster dataset was obtained from www.worldpop.org. The dataset was adjusted to match the corresponding UNPD estimate at the country level and had a spatial resolution of 100 x 100-m cell size grids. The study area's map was extracted from the dataset, resampled to 10 x 10-m cell size grids, and reclassified into five classes using the Natural Breaks standard classification scheme in ArcGIS 10.7.1. The categorization assigned values 1 through 5 to different hazard ratings based on increasing population count. Lower population count categories were assigned lower values due to their low susceptibility to flooding, while higher population count categories were assigned higher values due to their high susceptibility as more people living in an area increase exposure to floods [20,67].

The population density of Ethiopia for the year 2020 was obtained from www.worldpop.org. The acquired raster dataset was calibrated to conform to the corresponding United Nations Population Division's estimates at the country level, and it possessed a spatial resolution of 1000 × 1000 m cell size grids. Subsequently, the study area was extracted from the dataset and subjected to resampling, whereby the initial grid-cell size was altered to 10 x 10-m cell size grids ArcGIS 10.7.1. Finally, a five-class Natural Breaks standard classification scheme was employed to reclassify the data set, and the outputs were evaluated from 1 (very low) to 5 (very high) population density. Lower population densities indicate a lower susceptibility to flooding, while higher population densities signify increased vulnerability [20,68].

The total number of people in Ethiopia per grid square broken down by gender (women) and age (less than 5 and greater than 65 years) spatial distribution of the year 2020 was obtained from www.worldpop.org. With the spatial resolution of 100 x 100-m cell size grids. Then extracted and resampled to 10 x 10-m cell size grids and reclassified into Natural Breaks 1(very low) to 5 (very high) to flooding in ArcGIS 10.7.1. Older people greater than 65 years of age, children less than 5 years, and women are less mobile and need more assistance more vulnerable to flooding [20,65,66,69,70].

The study area's unemployment population, categorized by age and gender, was obtained from ACA. By applying the "Calculate Density" tool within ArcGIS 10.7.1 and a newly added field in the attribute table, a density map of the study area was generated from input point features. The resulting map was then resampled to 10 x 10-m cell-size grids and classified into five discrete classes based on Natural Breaks at increasing levels of unemployment. Lower unemployment levels indicate less coping with flood risk, while higher unemployment levels highlight heightened vulnerability [20,29,71].

The study area's slope map was generated using DEM with 12.5 m size grids, downloaded from ASTER- Global (http://earthexplorer.usgs.gov/) acquired in February 2023 and resampled to 10 x 10-m cell size grids. The spatial analysis tool slope in percentage ranges was developed using ArcGIS, 10.7.1, and a standard classification scheme called Natural Breaks was applied to classify the slope into five classes. The classification considered the susceptibility of the slope to flooding and assigned each value to a specific hazard rating. The susceptibility decreases from the lowest slope category, which was assigned a value of 5, to the highest slope category with a value of 1. The flatter topography, associated with lower slope values, was particularly vulnerable to flooding, whereas steeper topography, represented by higher slope values, was less vulnerable to flooding [72,73].

The productivity of the study area was mapped from the Normalized Difference in Vegetation Index (NDVI). A Sentinel 2 A satellite image, acquired from the USGS website (http://earthexplorer.usgs.gov/.accessed in February 2023) was utilized to generate the NDVI map. Equation (2) was applied in the ArcGIS (version 10.7.1) environment to calculate the NDVI. The NDVI values were resampled to 10 x 10-m cell size grids and classified into five Natural Breaks of susceptibility to flooding from very low to very high. Higher NDVI values indicate healthier vegetation cover, which reduces flood vulnerability by absorbing water and reducing runoff [[74], [75], [76]].In line with this, the NDVI map was produced based on equation (1).

(1) $$NDVI=\frac{NIR-RED}{NIR+RED}=\frac{Band\;8-Band\;4}{Band\;8+Band\;4}$$

The fractional impervious surface (FIS) of the study area was derived from the NDVI of the study area. Based on Skougaard Kaspersen, Fensholt [77], equations (2), (3), (4) were applied in ArcGIS 10.7.1 spatial analyst tools, map algebra, and raster calculator. Then resampled to 10 x 10-m cell size grids and classified into five classes based on Natural Breaks. High impervious surface fractions increase the risk of flooding by reducing the area available for water to infiltrate and increasing the speed of runoff [77].

(2) $$NDVI\left(S\right)=\frac{NDVI-NDVI\left(Low\right)}{NDVI\left(High\right)-NDVI\left(Low\right)}$$

(3) FVC(FractionalVegetationCover)=(NDVI(S))²

(4) FIS(FractionalImperviousSurface)=1−FVC

To map the proximity of ecosystems to potential toxic industries, shapefiles of potential toxic industries were obtained from ACA and analysis tools from ArcGIS 10.7.1 were utilized to create multiple ring buffers within a given distance for the study area. The resulting index was then subjected to a union process to identify all ecosystems that intersect with potentially toxic industries. The obtained map of ecosystems and potential toxic industry locations were then resampled to 10 x 10-m cell size grids and classified into five categories based on Natural Breaks to identify areas at risk of exposure to toxic substances during floods. Ecosystems near toxic industries are more vulnerable to flooding than further areas due to the potential exposure to toxic substances during floods as it exposes nearby communities to toxic chemicals and hazardous waste [7,15,78].

To map the index and average patch size of the study vegetation layer from ACA were derived and digitized to update the layer digitizing based on field visit and high-resolution Google Earth Pro-Satellite imagery (Google Earth Pro_Version 7.3.6). Then, patch sizes of vegetation layers were classified based on their area. Then resampled to 10 x 10-m cell size grids and classified into five classes based on Natural Breaks in ArcGIS 10.7.1. The size of the patches determines areas that are vulnerable to flooding. Smaller patches indicate areas that are more fragmented and less able to provide ecosystem services that help mitigate flood impacts [79,80].

The bare soil raster dataset was obtained from the Water and Land Resource Center (WLRC), Ethiopia, and the wetland of the study area was obtained from ACA. Then, resampled to 10 x 10-m cell size grids and computed into percentages in ArcGIS 10.7.1. Bare-soil areas are more vulnerable to flooding due to their low infiltration capacity and high erodibility [15] and whereas wetlands absorb flood water and reduce flooding [81].

The built-up area of the study area was extracted from an ESRI website: https://livingatlas.arcgis.com/Land cover/(acquired in February 2023) with 10 x 10-m cell size grids. Then, computed into percentages in an ArcGIS 10.7.1 environment. Built-up areas are more vulnerable to flooding due to their inability to percolate water into the subsurface, which makes the area more vulnerable [82].

A road density map was created using a road shapefile acquired from ACA, with any missing data supplemented by digitizing based on field visits and high-resolution Google Earth Pro-Satellite imagery (Google Earth ProVersion 7.3.6). Kernel line density tools were utilized to derive linear feature density, which was then resampled to 10 x 10-m cell size grids and classified into five classes based on Natural Breaks. High road density increases the risk of flooding by reducing the area available for water to infiltrate and increasing the speed of runoff [83,84]. A green infrastructure density map was similarly generated using a corresponding GI shapefile, with its polygon features converted to point form before kernel point density was computed in ArcGIS 10.7.1. This resulting GI density map was also resampled and classified in the same manner as that of the road density map. Green infrastructure reduces the vulnerability of an area to flooding by increasing infiltration and reducing runoff [85,86].

Critical infrastructure (potential toxic industries, landfill sites, abattoirs, gas terminals) that affect public health and safety shapefiles were obtained from ACA was obtained from ACA and analysis tools from ArcGIS 10.7.1 was utilized to create multiple ring buffers within a given distance for the study area. The resulting map was then resampled to 10 x 10-m cell-size grids and classified into five classes based on Natural Breaks. Critical infrastructure such as toxic industries, landfill sites, abattoirs, and gas terminals are exposed to floods and important to identify the areas from the point sources [[87], [88], [89]].

Emergency centers (hospitals, schools, universities, and community centers) shapefiles were obtained from ACA and updated the missing emergency centers through digitizing based on field visits and high-resolution Google Earth Pro-Satellite imagery (Google Earth Pro version 7.3.6). Then, analysis tools from ArcGIS 10.7.1 were utilized to create multiple ring buffers within a given distance for the study area. The resulting map was then resampled to 10 x 10-m cell-size grids and classified into five classes based on Natural Breaks. Emergency centers such as hospitals, schools, and universities play a critical role in assisting the community during floods and serve as emergency response sites [90,91].

#### 2.3.2 Method of analytical hierarchy processing (AHP)

The AHP techniques developed by Saaty [92] were used for determining the priorities of multiple criteria decision problems in characterizing flood vulnerability. In urban flood vulnerability analysis, AHP was used for multi-criteria decisions by many researchers [[93], [94], [95], [96]].

AHP analysis was implemented to quantify the comparative significance of flood-controlling variables. In this process, a matrix of pairwise comparisons was computed (Table 5), followed by its normalization and weighted assignment of the factors (Table 6). To check the consistency, a further evaluation was carried out (Table 7) following the reclassification of each variable as presented in Fig. 6(a–l) and Fig. 7(a–f).

As suggested by Saaty [92] the succeeding procedures were used to determine relative weights to each flood-conditioning factor including.

1. The matrix of pair-wise comparisons is constructed based on Saaty's 1 to 9 scale, where each variable was represented in Table 2.

**Table 2:** Saaty's pair-wise comparison scale.

Intensity of importance	Degree of preference	Explanation
1	Equal importance	Two elements contribute equally to the objective
3	Moderate importance	Experience and judgment slightly favor one parameter over another
5	The strong or essential importance	Experience and judgment strongly favor one activity over another
7	Very strong importance	One parameter is favored very strongly and is considered superior to another; its dominance is demonstrated in practice
9	Extreme importance	The evidence favoring one parameter as superior to another is of the highest possible order of affirmation
2,4,6,8	Intermediate values	When parameters that are very close in importance

2. Then, each entry in the given matrix of pair-wise comparisons column was divided by its corresponding sum (refer to Table 5)
3. Finally, each weight was determined by dividing the total of each line in the normalized matrix by eighteen (refer to Table 5).

After calculating the weights of each variable related to flood vulnerability characterization, a consistency index check was computed using equation (5) [92], if the comparison is accurate.

(5) $$CI=\frac{\lambda_{\max-n}}{n-1}$$

Where; 'CI' denotes the consistency index, 'n' stands for the number of factors being examined, and ‘λ’ max represents the maximum eigenvalue of the comparison pairwise matrix.

The following steps were used to compute the maximum Eigenvalue (λmax) of the comparison pair-wise matrix [92].

1. Multiplying each column value (in the non-normalized matrix) by the weight criteria
2. The rows' values are added to calculate the weighted sum
3. Weighting each criterion value based on its weighted sum value, and
4. A weighted sum of the mean concerning the criteria weights.

Then, the calculated consistency ratio (CR) which demonstrates the validity of Saaty [92] comparison was computed using Equation (6).

(6) $$CR=\frac{CI}{RI}$$

According to Saaty [92], if the consistency ratio is less than 0.10, the matrix of pair-wise comparisons is considered consistent, however, if it is greater or equal to 0.10, insufficient consistency is present and the procedure must be iterated several times until the CR value drops below this threshold. Equation (3) presented the random consistency index to the matrix size (Table 3).

**Table 3:** Random consistency index (RCI).

**Size of matrix**	1	2	3	4	5	6	7	8	9	10	11	12	13	14	15	16	17	18
**RCI**	**0**	**0**	**0.58**	**0.9**	**1.12**	**1.24**	**1.32**	**1.41**	**1.45**	**1.49**	**1.51**	**1.48**	**1.56**	**1.57**	**1.59**	**1.605**	**1.61**	**1.615**

#### 2.3.3 Method of Preparing the SET vulnerability map

To develop the flood vulnerability map of the study area, all the selected SET variables were geo-referenced with the same coordinate system of WGS_1984_UTM_Zone_37 N, resampled to 10 x 10-m cell size grids, reclassified, and weighted via Analytic Hierarchy. Afterward, ArcGIS's Spatial Analyst extension's weighted overlay technique was employed, with Equation (7). This equation has been employed in numerous prior studies for assessing areas posed with a heightened risk of flooding [84,93,95]. Accordingly, the vulnerability maps for the seven classes (i.e., S, E, T, S-E, S-T, E-T, S-E-T) were mapped.

(7) $$FP=\sum_{i=0}^{n}Xi*Wi$$

Where; ‘*FP’* denotes flood vulnerability, ‘*n’ is the* number of decision-making criteria, ‘*Xi’ is the* specific normalized criterion, and ‘*WI’ is* the criterion's weight.

#### 2.3.4 Methods of analyzing the spatial distribution of vulnerability maps

Global Moran's I to identify if the spatial patterns of flood vulnerability were clustered, dispersed, or random in ArcGIS 10.7.1 Moran's I value close to zero indicates random spatial distribution, while positive and negative values indicate clustering (similar neighborhoods are next to each other) or dispersed (dissimilar neighborhoods are next to each other) spatial patterns, respectively [15,98].

#### 2.3.5 Method of validation of the SETS map

Validation is a crucial step in ensuring that model outputs accurately portray real-world conditions on the ground. For this study, field-based visits and interviews with local dwellers were conducted both before and after modeling to enhance understanding and precision. In addition, ACA experts were interviewed, and historical flood inundation data were cross-checked with satellite imagery from Google Earth Pro Version 7.3.6. Furthermore, the final SETS map was compared with 120 Ground Control Points (GCPs) collected using a handheld Global Positioning System (GPS) device (Model: BHCNav6). The GCPs were then converted to shapefiles, overlaid onto the final SETS map on ArcGIS version 10.7.1 for verification, and proved the model precision.

### 2.4 Limitations of the adopted methodology

The Social-Ecological-Technological (SET) approach to urban flood vulnerability assessment has been proven as a comprehensive method that utilizes multi-criteria decision-making techniques. However, this approach has certain limitations, such as the absence of climate change projections and scenarios, uncertainty, and risk perception.

## 3 Result

### 3.1 SET variables

The eighteen SET variables with their unit, classes, flood susceptibility, rating values, and weight of the study area as analyzed using ArcGIS 10.7.1 Multi-Criteria Decision-Making and Analytical Hierarchy Process is presented in Table 4.

**Table 4:** SETS variables their abbreviation (Abb.), unit, classes, flood susceptibility, rating values, and weight.

No	Variables	Abbreviation	Unit	Class	Flood susceptibility	Susceptibility rating	Weight (%)
1	Total population count	PT	total number of people per grid-cell	0.45–8.73	Very low	1	6.08
				8.73–35.36	Low	2	
				35.36–74.40	Medium	3	
				74.40–109.31	High	4	
				109.31–151.39	Very high	5	
2	Population density	PD	# of people/area	57 - 1323	Very low	1	6.77
				1323 - 4208	Low	2	
				4208 - 8289	Medium	3	
				8289 - 12,792	High	4	
				12,792 - 17,999	Very high	5	
3	Percentage of children <5 years	PC	%	0.04–0.25	Very low	1	6.64
				0.25–0.58	Low	2	
				0.58–1.00	Medium	3	
				1.00–1.43	High	4	
				1.43–2.25	Very high	5	
4	Percentage of the population over 65 years	PO	%	0.02–0.12	Very low	1	6.13
				0.12–0.30	Low	2	
				0.30–0.52	Medium	3	
				0.52–0.75	High	4	
				0.75–1.17	Very high	5	
5	Percentage of women	PW	%	0.0094–0.062	Very low	1	6.70
				0.063–0.15	Low	2	
				0.16–0.26	Medium	3	
				0.27–0.38	High	4	
				0.39–0.59	Very high	5	
6	Percentage of unemployed people	PU	%	0.03–0.15	Very low	1	6.25
				0.15–4.22	Low	2	
				4.22–11.49	Medium	3	
				11.49–18.26	High	4	
				18.26–31.45	Very high	5	
7	Slope variation	S	%	0–3.14	Very high	5	8.49
				3.14–7.12	High	4	
				7.12–13.21	Medium	3	
				13.21–22.21	Low	2	
				22.22–53.42	Very low	1	
8	The proximity of the ecosystem to toxic release sites	PE	Level	1–3	Very low	1	7.04
				3–4	Low	2	
				4–5	Medium	3	
				5–6	High	4	
				6–7	Very high	5	
9	Combination of shape index and average patch size	SIPS	Area(m^2^)	0.0034–22.77	Very high	5	6.78
				22.77–50.90	High	4	
				50.91–99.13	Medium	3	
				99.13–136.64	Low	2	
				136.65–341.61	Very low	1	
10	Percentage of bare soil within the area	BS	%	2.32	Very high	5	5.49
11	Percentage of wetlands within the area	W	%	0.83	Very low	1	5.32
12	Productivity based on Normalized Difference in Vegetation Index (NDVI)	PNDVI	Level	−0.16–0.098	Very high	5	4.35
				0.099–0.13	High	4	
				0.14–0.16	Medium	3	
				0.17–0.23	Low	2	
				0.24–0.46	Very low	1	
13	Building area (percentage of building area within the area)	BA	%	25.50	High	4	4.36
14	Critical infrastructure (CI) (wastewater, polluted industries, gas terminal) in the area	CI	Meter(m)	<100	Very high	5	4.38
				100–500	High	4	
				500 - 1000	Medium	3	
				1000–3000	Low	2	
				>3000	Very low	1	
15	Road density	RD	Length of road/area	0–2.07	Very low	1	4.21
				2.07–6.31	Low	2	
				6.31–12.86	Medium	3	
				12.87–19.41	High	4	
				19.41–29.28	Very high	5	
16	Fractional Impervious surface	FIS	%	−0.0000012–0.61	Very low	1	3.49
				0.62–0.77	Low	2	
				0.78–0.87	Medium	3	
				0.88–0.92	High	4	
				0.93–1	Very high	5	
17	Green Infrastructure (GI) density	GI	Number of GI/area	0–26.57	Very high	5	3.46
				26.57–75.64	High	4	
				75.64–145.15	Medium	3	
				145.16–265.77	Low	2	
				265.78–521.31	Very low	1	
18	Emergency centers (distance of emergency centers (e.g., hospitals, schools, community centers))	EC	Meter (m)	<500	Very low	1	4.05
				500–1500	Low	2	
				1500–2500	Medium	3	
				2500–5000	High	4	
				>5000	Very high	5	
Total							100.00

Table 4 shows the SET variables, unit, classes, susceptibility, rating values, and weight. The AHP model utilized the pair-wise comparison matrix (shown in Table 5) to determine the prioritization of these factors, with each factor's weight represented as a percentage value ranging from 0 to 100 %. The weight and ranking of each factor were calculated by employing both the pair-wise comparison matrix and the factor map. The weight value assigned to each factor indicated its prioritization and was expressed as a percentage value between 0 and 100 %. The sum of all weights equaled 100 % due to the use of a linear weighted combination.

**Table 5:** Pair-wise comparison matrix.

Variables	PT	PD	PC	PO	PW	PU	S	PE	SIPS	BS	W	PNDVI	BA	CI	RD	FIS	GI	EC
PT	1	1	1	1	1	1	1	2	1	1	1	1	2	1	1	2	1	2
PD	1	1	1	2	1	2	1	1	1	2	2	2	2	1	1	1	1	1
PC	0	1	1	1	1	2	2	2	1	1	1	2	2	2	1	2	1	1
PO	1	0	1	1	1	2	2	2	2	1	1	1	1	1	1	1	1	1
PW	1	1	0	1	1	1	2	2	2	2	2	2	2	1	1	1	1	1
PU	1	1/2	1/2	0	1	1	1	2	2	1	1	2	3	2	2	1	2	1
S	1	1	1/2	1/2	0	1	1	2	3	3	2	3	3	2	3	3	3	2
PE	1/2	1	1/2	1/2	1/2	0	1/2	1	1	2	3	2	3	3	3	3	3	2
SIPS	1	1	1	1/2	1/2	1/2	0	1	1	2	3	3	2	1	1	3	3	3
BS	1	1/2	1	1	1/2	1	1/3	0	1/2	1	2	2	2	2	2	2	2	1
W	1	1/2	1	1	1/2	1	1/2	1/3	0	1/2	1	3	2	2	2	2	2	1
PNDVI	1	1/2	1/2	1	1/2	1/2	1/3	1/2	1/3	0	1/3	1	1	2	2	2	2	2
BA	1/2	1/2	1/2	1	1/2	1/3	1/3	1/3	1/2	1/2	0	1	1	2	3	2	2	2
CI	1	1	1/2	1	1	1/2	1/2	1/3	1	1/2	1/2	0	1/2	1	2	2	2	1
RD	1	1	1	1	1	1/2	1/3	1/3	1	1/2	1/2	1/2	0	1/2	1	2	2	1
FIS	1/2	1	1/2	1	1	1	1/3	1/3	1/3	1/2	1/2	1/2	1/2	0	1/2	1	2	1
GI	1	1	1	1	1	1/2	1/3	1/3	1/3	1/2	1/2	1/2	1/2	1/2	0	1/2	1	1
EC	1/2	1	1	1	1	1	1/2	1/2	1/3	1	1	1/2	1/2	1	1	0	1	1
Sum	15.00	14.50	13.50	16.50	14.00	16.83	14.00	18.00	18.33	20.00	22.33	27.00	28.00	25.00	27.50	30.50	32.00	25.00

**Table 6:** Normalized pair-wise comparison matrix.

Variables	PT	PD	PC	PO	PW	PU	S	PE	SIPS	BS	W	PNDVI	BA	CI	RD	FIS	GI	EC	Sum	WSV	CW (%)
**PT**	0.0667	0.0690	0.0741	0.0606	0.0714	0.0594	0.0714	0.1111	0.0545	0.0500	0.0448	0.0370	0.0714	0.0400	0.0364	0.0656	0.0313	0.0800	**1.0947**	**0.0608**	**6.08**
**PD**	0.0667	0.0690	0.0741	0.1212	0.0714	0.1188	0.0714	0.0556	0.0545	0.1000	0.0896	0.0741	0.0714	0.0400	0.0364	0.0328	0.0313	0.0400	**1.2181**	**0.0677**	**6.77**
**PC**	0.0000	0.0690	0.0741	0.0606	0.0714	0.1188	0.1429	0.1111	0.0545	0.0500	0.0448	0.0741	0.0714	0.0800	0.0364	0.0656	0.0313	0.0400	**1.1959**	**0.0664**	**6.64**
**PO**	0.0667	0.0000	0.0741	0.0606	0.0714	0.1188	0.1429	0.1111	0.1091	0.0500	0.0448	0.0370	0.0357	0.0400	0.0364	0.0328	0.0313	0.0400	**1.1026**	**0.0613**	**6.13**
**PW**	0.0667	0.0690	0.0000	0.0606	0.0714	0.0594	0.1429	0.1111	0.1091	0.1000	0.0896	0.0741	0.0714	0.0400	0.0364	0.0328	0.0313	0.0400	**1.2056**	**0.0670**	**6.70**
**PU**	0.0667	0.0345	0.0370	0.0000	0.0714	0.0594	0.0714	0.1111	0.1091	0.0500	0.0448	0.0741	0.1071	0.0800	0.0727	0.0328	0.0625	0.0400	**1.1247**	**0.0625**	**6.25**
**S**	0.0667	0.0690	0.0370	0.0303	0.0000	0.0594	0.0714	0.1111	0.1636	0.1500	0.0896	0.1111	0.1071	0.0800	0.1091	0.0984	0.0938	0.0800	**1.5276**	**0.0849**	**8.49**
**PE**	0.0333	0.0690	0.0370	0.0303	0.0357	0.0000	0.0357	0.0556	0.0545	0.1000	0.1343	0.0741	0.1071	0.1200	0.1091	0.0984	0.0938	0.0800	**1.2679**	**0.0704**	**7.04**
**SIPS**	0.0667	0.0690	0.0741	0.0303	0.0357	0.0297	0.0000	0.0556	0.0545	0.1000	0.1343	0.1111	0.0714	0.0400	0.0364	0.0984	0.0938	0.1200	**1.2209**	**0.0678**	**6.78**
**BS**	0.0667	0.0345	0.0741	0.0606	0.0357	0.0594	0.0238	0.0000	0.0273	0.0500	0.0896	0.0741	0.0714	0.0800	0.0727	0.0656	0.0625	0.0400	**0.9879**	**0.0549**	**5.49**
**W**	0.0667	0.0345	0.0741	0.0606	0.0357	0.0594	0.0357	0.0185	0.0000	0.0250	0.0448	0.1111	0.0714	0.0800	0.0727	0.0656	0.0625	0.0400	**0.9583**	**0.0532**	**5.32**
**PNDVI**	0.0667	0.0345	0.0370	0.0606	0.0357	0.0297	0.0238	0.0278	0.0182	0.0000	0.0149	0.0370	0.0357	0.0800	0.0727	0.0656	0.0625	0.0800	**0.7825**	**0.0435**	**4.35**
**BA**	0.0333	0.0345	0.0370	0.0606	0.0357	0.0198	0.0238	0.0185	0.0273	0.0250	0.0000	0.0370	0.0357	0.0800	0.1091	0.0656	0.0625	0.0800	**0.7855**	**0.0436**	**4.36**
**CI**	0.0667	0.0690	0.0370	0.0606	0.0714	0.0297	0.0357	0.0185	0.0545	0.0250	0.0224	0.0000	0.0179	0.0400	0.0727	0.0656	0.0625	0.0400	**0.7892**	**0.0438**	**4.38**
**RD**	0.0667	0.0690	0.0741	0.0606	0.0714	0.0297	0.0238	0.0185	0.0545	0.0250	0.0224	0.0185	0.0000	0.0200	0.0364	0.0656	0.0625	0.0400	**0.7587**	**0.0421**	**4.21**
**FIS**	0.0333	0.0690	0.0370	0.0606	0.0714	0.0594	0.0238	0.0185	0.0182	0.0250	0.0224	0.0185	0.0179	0.0000	0.0182	0.0328	0.0625	0.0400	**0.6285**	**0.0349**	**3.49**
**GI**	0.0667	0.0690	0.0741	0.0606	0.0714	0.0297	0.0238	0.0185	0.0182	0.0250	0.0224	0.0185	0.0179	0.0200	0.0000	0.0164	0.0313	0.0400	**0.6234**	**0.0346**	**3.46**
**EC**	0.0333	0.0690	0.0741	0.0606	0.0714	0.0594	0.0357	0.0278	0.0182	0.0500	0.0448	0.0185	0.0179	0.0400	0.0364	0.0000	0.0313	0.0400	**0.7283**	**0.0405**	**4.05**
Total																					**100**

Where: *WSV* weighted sum value, *CW* criteria weight.

**Table 7:** Calculating the consistency of pairwise comparison (**CR = 0.05**).

variables	PT	PD	PC	PO	PW	PU	S	PE	SIPS	BS	W	PNDVI	BA	CI	RD	FIS	GI	EC
PT	0.060814506	0.0677	0.0664	0.0613	0.0670	0.0625	0.0849	0.1409	0.0678	0.0549	0.0532	0.0435	0.0873	0.0438	0.0421	0.0698	0.0346	0.0809
PD	0.060814506	0.0677	0.0664	0.1225	0.0670	0.1250	0.0849	0.0704	0.0678	0.1098	0.1065	0.0869	0.0873	0.0438	0.0421	0.0349	0.0346	0.0405
PC	0	0.0677	0.0664	0.0613	0.0670	0.1250	0.1697	0.1409	0.0678	0.0549	0.0532	0.0869	0.0873	0.0877	0.0421	0.0698	0.0346	0.0405
PO	0.060814506	0.0000	0.0664	0.0613	0.0670	0.1250	0.1697	0.1409	0.1357	0.0549	0.0532	0.0435	0.0436	0.0438	0.0421	0.0349	0.0346	0.0405
PW	0.060814506	0.0677	0.0000	0.0613	0.0670	0.0625	0.1697	0.1409	0.1357	0.1098	0.1065	0.0869	0.0873	0.0438	0.0421	0.0349	0.0346	0.0405
PU	0.060814506	0.0338	0.0332	0.0000	0.0670	0.0625	0.0849	0.1409	0.1357	0.0549	0.0532	0.0869	0.1309	0.0877	0.0843	0.0349	0.0693	0.0405
S	0.060814506	0.0677	0.0332	0.0306	0.0000	0.0625	0.0849	0.1409	0.2035	0.1646	0.1065	0.1304	0.1309	0.0877	0.1264	0.1048	0.1039	0.0809
PE	0.030407253	0.0677	0.0332	0.0306	0.0335	0.0000	0.0424	0.0704	0.0678	0.1098	0.1597	0.0869	0.1309	0.1315	0.1264	0.1048	0.1039	0.0809
SIPS	0.060814506	0.0677	0.0664	0.0306	0.0335	0.0312	0.0000	0.0704	0.0678	0.1098	0.1597	0.1304	0.0873	0.0438	0.0421	0.1048	0.1039	0.1214
BS	0.060814506	0.0338	0.0664	0.0613	0.0335	0.0625	0.0283	0.0000	0.0339	0.0549	0.1065	0.0869	0.0873	0.0877	0.0843	0.0698	0.0693	0.0405
W	0.060814506	0.0338	0.0664	0.0613	0.0335	0.0625	0.0424	0.0235	0.0000	0.0274	0.0532	0.1304	0.0873	0.0877	0.0843	0.0698	0.0693	0.0405
PNDVI	0.060814506	0.0338	0.0332	0.0613	0.0335	0.0312	0.0283	0.0352	0.0226	0.0000	0.0177	0.0435	0.0436	0.0877	0.0843	0.0698	0.0693	0.0809
BA	0.030407253	0.0338	0.0332	0.0613	0.0335	0.0208	0.0283	0.0235	0.0339	0.0274	0.0000	0.0435	0.0436	0.0877	0.1264	0.0698	0.0693	0.0809
CI	0.060814506	0.0677	0.0332	0.0613	0.0670	0.0312	0.0424	0.0235	0.0678	0.0274	0.0266	0.0000	0.0218	0.0438	0.0843	0.0698	0.0693	0.0405
RD	0.060814506	0.0677	0.0664	0.0613	0.0670	0.0312	0.0283	0.0235	0.0678	0.0274	0.0266	0.0217	0.0000	0.0219	0.0421	0.0698	0.0693	0.0405
FIS	0	0.0677	0.0332	0.0613	0.0670	0.0625	0.0283	0.0235	0.0226	0.0274	0.0266	0.0217	0.0218	0.0000	0.0211	0.0349	0.0693	0.0405
GI	0	0.0677	0.0664	0.0613	0.0670	0.0312	0.0283	0.0235	0.0226	0.0274	0.0266	0.0217	0.0218	0.0219	0.0000	0.0175	0.0346	0.0405
EC	0.030407253	0.0677	0.0664	0.0613	0.0670	0.0625	0.0424	0.0352	0.0226	0.0549	0.0532	0.0217	0.0218	0.0438	0.0421	0.0000	0.0346	0.0405

**Fig. 3:**
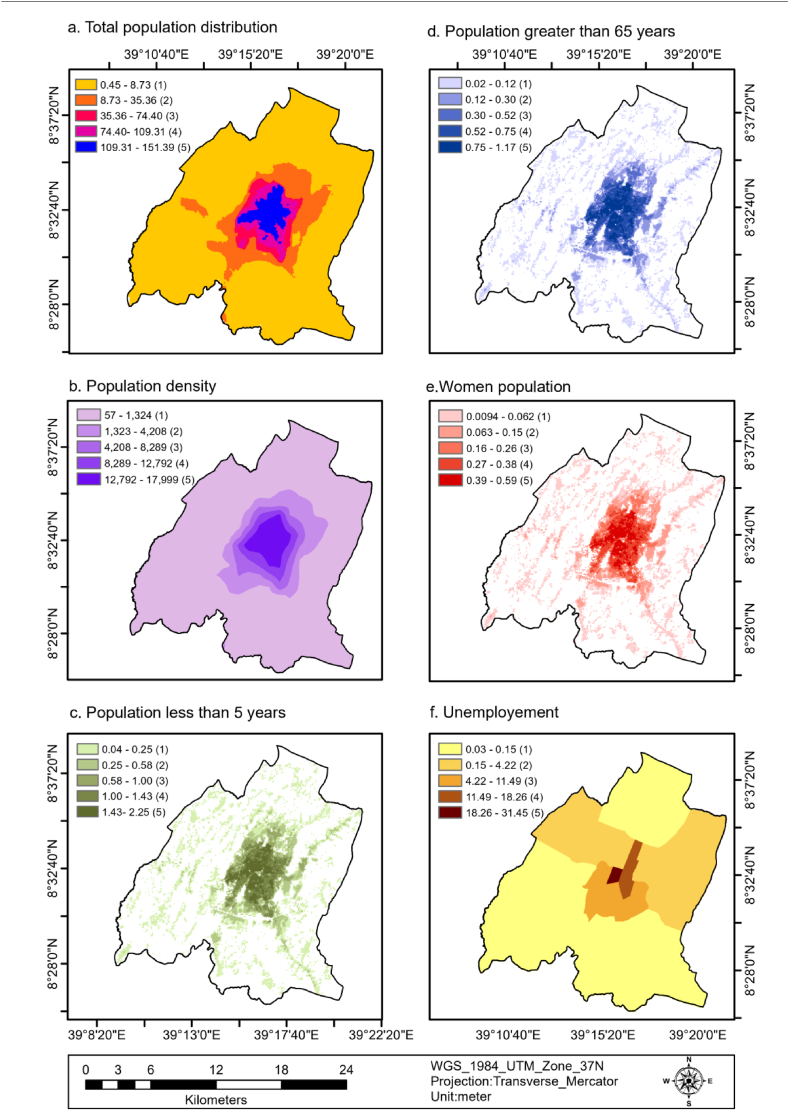
Total population distribution (a), population density (b), population <5 years(c), population >65 years (d), women population (e), unemployment (f).

The spatial distribution of the population count in the study area is represented as rasterized data, which varies between 0.45 and 151.39. The data is divided into five categories based on these values: 0.45–8.73 (very low), 8.73–35.36 (low), 35.36–74.40 (medium), 74.40–109.31 (high), and 109.31–151.39 (very high). These categories cover 78.96 %, 11.75 %, 2.64 %, 3.33 %, and 3.32 % of the population respectively, as shown in Fig. 3 (a) and Table 4. Based on these findings, around 9.29 % of the population residing in the study region has moderate to high vulnerability to flooding. This implies that the individuals belonging to this particular percentage of the overall population of the study area were prone to the risk of flooding due to their settlements.

The population density of the study area ranges from 56.81 to 17,998.16 and is categorized into five classes 56.81–1323.26 (very low), 1323.26–4207.94 (low), 4207.94–8288.72 (medium), 8288.72–12,791.64 (high) and 12,791.64–17,998.16 (very high) which covers 79.53 %, 9.38 %, 4.16 %, 3.18 %, and 3.75 %, as shown in Fig. 3 (b) and Table 4. This indicates that about 11.09 % of the population of the study area is moderate to very highly vulnerable to flooding as a result of being densely populated.

The population of children less than five years was ranges from 0.04 to 2.25 and categorized into five classes 0.04–0.25 (very low), 0.25–0.58 (low), 0.58–1.00 (medium), 1.00–1.43 (high), and 1.43–2.25 (very high) which covers 54.37 %, 18.37 %, 9.18 %, 10.67 %, 7.41 %, as indicated in Fig. 3 (c) and Table 4. This implies that about 27.26 % children population is vulnerable to flooding during flooding in the study area.

The population of people greater than 65 years was ranges from 0.02 to 1.17 and categorized into five classes 0.02–0.12 (very low), 0.12–0.30 (low), 0.30–0.52 (medium), 0.52–0.75 (high), 0.75–1.17 (very high) which covers 54.95 %, 17.99 %, 9.06 %, 10.73 %, and 7.26 % respectively, as indicated in Fig. 3 (d) and Table 4. This implies that about 27.05 of the elder population is vulnerable to flooding during flooding.

The women population of the study area ranges from 0.0094 to 0.59 and is categorized into five classes 0.0094–0.062 (very low), 0.063–0.15(low), 0.16–0.26 (medium), 0.27–0.38 (high), and 0.39–0.59 (very high) which covers 55.17 %, 17.75 %, 8.84 %, 10.69 %, and 7.55 %, as indicated in Fig. 3 (e) and Table 4. This implies that about 27.08 % of the women population is vulnerable to flooding.

The unemployment population density of the study area ranges from 0.03 to 31.45 and is categorized into five classes 0.03–0.15 (very low), 0.15–4.22 (low), 4.22–11.49 (medium), 11.49–18.26 (high), and 18.26–31.45 (very high) which covers 63.9, 28.05, 5.98, 1.65, and 0.43, as indicated in Fig. 3 (f) and Table 4. About 8.06 % of the population is moderately to very highly vulnerable to flooding due to unemployment.

**Fig. 4:**
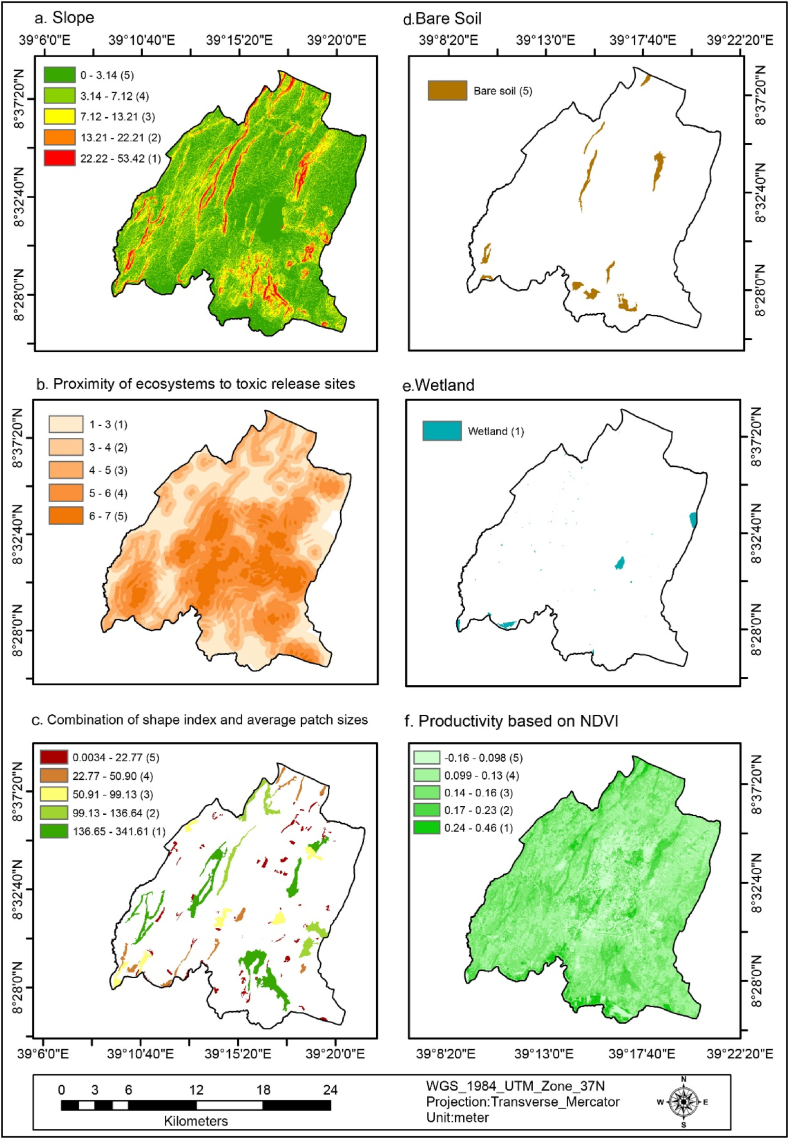
Slope(a), the proximity of ecosystems to toxic release sites (b), the combination of shape index and average patch sizes(c), bare soil (d), wetland(e), productivity based on NDVI (f).

The slope of the study area was classified into five classes 0–3.14°(very high), 3.14–7.12°(high), 7.12–13.21°(medium), 13.21–22.21°(low), and 22.22–53.42°(very low) which covers 2.08 %, 5.32 %, 14.38 %, 38.21 %, and 40 % of the total area, as indicated in Fig. 4 (a) and Table 4. This implies that about 92.59 % of the area covers moderate to very high flood vulnerability. The implication of this is that the study area's gentle slopes (flat surfaces) triggered flooding.

The Proximity of the ecosystem to toxic release areas was classified into five classes 1–3 (very low), 3–4 (low), 4–5 (medium), 5–6 (high), and 6–7(very high) which covers 21.4 %, 23.51 %, 19.43 %, 24.84 %, 10.82 % respectively, as indicated in Fig. 4 (b) and Table 4. This implies that about 55.09 % of the ecosystem has a medium to very high susceptibility to flooding. Therefore, over half of the ecosystems are near sites where harmful substances may be released and are at a greater risk of flooding during inundation.

The combination of shape index and average patch sizes are categorized into five classes 0.0034–22.77 (very high), 22.77–50.90 (high), 50.91–99.13(medium), 99.13–136.64 (low), and 136.65–341.61 (very low) which covers 42.76 %, 18.95 %, 14.26 %, 10.87 %, and 13.16 %, as shown in Fig. 4 (c) and Table 4. This implies that about 38.73% of shape index and patch sizes are medium to very highly vulnerable to flooding. The implication of this is that the small size shape index and scattered patches are vulnerable to flooding.

**Fig. 5:**
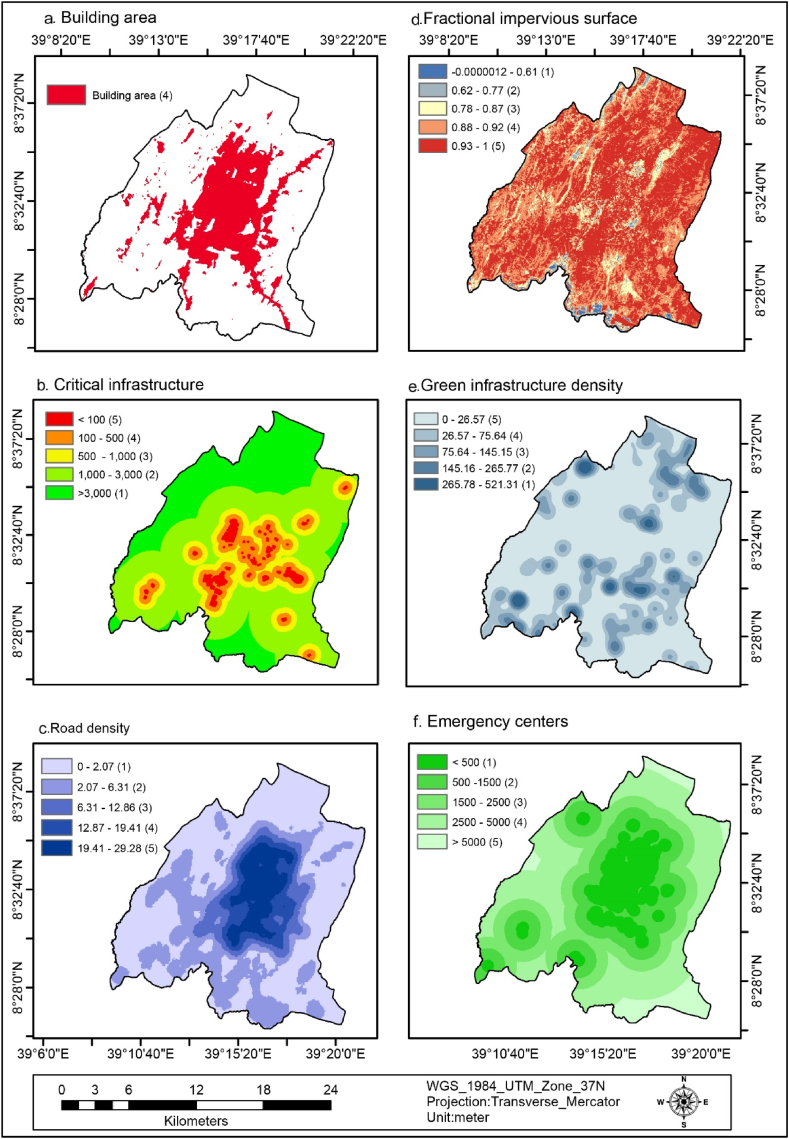
Building area (a), critical infrastructure (b), road density (c), FIS (d), GI density (e), and emergency centers(f).

The percentage of the bare soil in the study area accounted for 2.3 % (718.74 ha) as indicated in Fig. 4 (d) and Table 4. This was reclassified as having a very high flood vulnerability due to limited water infiltration, which facilitates the flow of floodwater across the surface. The built area covered 25.23 % (7871.49 ha) of the study area, as depicted in Fig. 5 (a) and Table 4, and was reclassified as having a high flood vulnerability. This is because structures and paved surfaces hinder water infiltration, leading to increased flooding. Furthermore, about 0.7 % (217.93 ha) of the study area consisted of wetlands, as shown in Fig. 4 (e) and Table 4. These wetlands were reclassified as having a very low flood vulnerability due to their capacity to retain significant amounts of water, thereby reducing the risk of flooding.

**Fig. 6:**
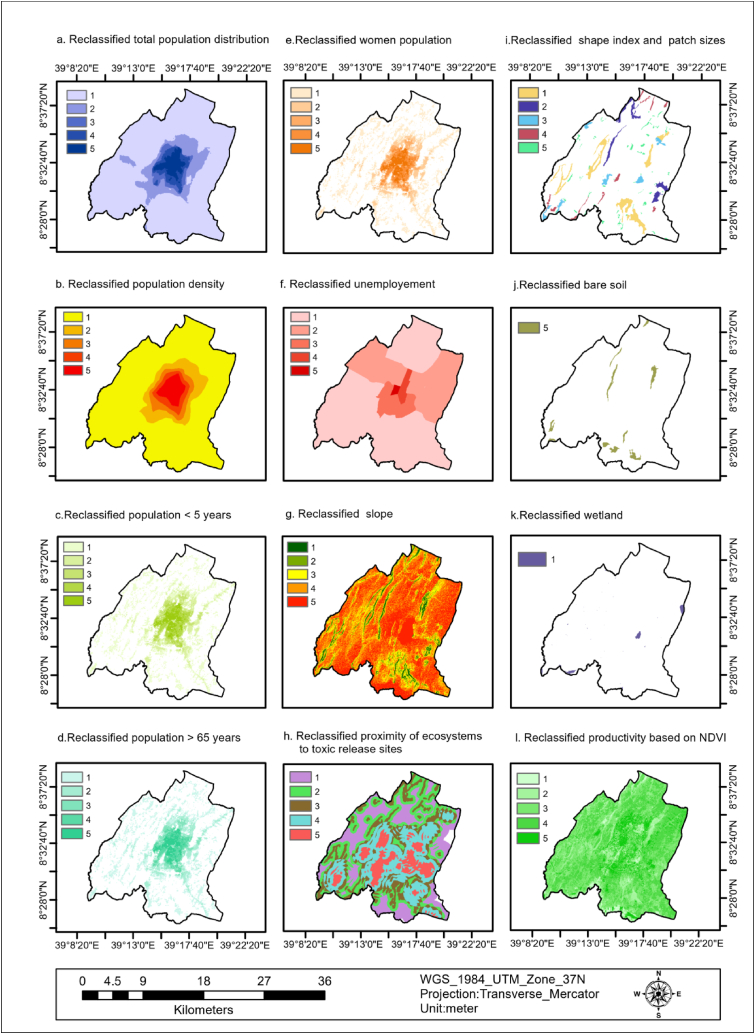
Reclassified SETS variables (a–l).

**Fig. 7:**
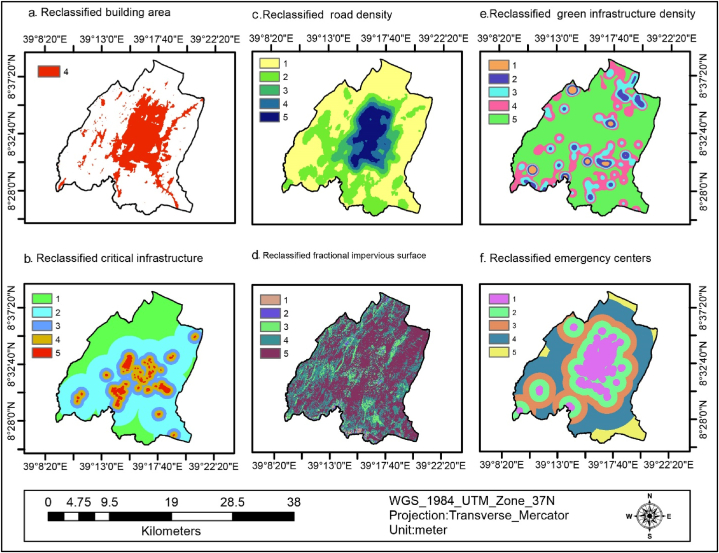
Reclassified SETS variables (a–f).

The productivity-based NDVI of the study area was classified into five classes −0.16 - 0.098 (very high), 0.099–0.13 (high), 0.14–0.16 (medium), 0.17–0.23 (low), and 0.24–0.46 (very low), which covers 7.54 %, 39.66 %, 38.68 %, 11.74 %, and 2.38 % of the study area respectively, as shown in Fig. 4 (f) and Table 4. This implies that about 85.88 % area was medium to very highly vulnerable to flooding. As a result of less healthy vegetation cover, increased runoff, and flood vulnerability prevail.

Critical Infrastructures((potential toxic industries, landfill site, abattoir, gas terminals) of the study area were buffered and categorized into five classes <100 m (very high), 100–500 m (high), 500 - 1000 m (medium), 1000–3000 (low), and >3000 (very low) which covers 3.41 %, 10.34 %, 13.54 %, 44.28 %, and 28.44 % respectively, as indicated in Fig. 5 (b) and Table 4. This implies that 27.29 % of the Critical infrastructure are found within moderate to very high flood vulnerability area. This implies that a quarter of the area is exposed to flooding due to the availability of nearby CI.

Road density of the study area was classified into five classes 0–2.07 (very low), 2.07–6.31(low), 6.31–12.86 (medium), 12.87–19.41(high), and 19.41–29.28 (very high) which covers 50.86%, 26.97 %, 7.13 %, 6.86 %, and 8.18 % of the study area respectively, as shown in Fig. 5 (c) and Table 4. This implies that 22.17 % of the area is moderate to very high flood vulnerability. This implies that more than a quarter of the road density increases the risk of flooding by reducing the area available for water to infiltrate and increasing the speed of runoff.

The fractional impervious surface of the study area is classified into five classes −0.0000012 - 0.61(very low), 0.62–0.77(low), 0.78–0.87(medium), 0.88–0.92 (high), and 0.93–1 (very high) which covers 0.67 %, 2.21 %, 8.86 %, 34.29 %, and 53.97 % respectively, as shown in Fig. 5 (d) and Table 4. This implies that about 97.12 of the area was moderate to very highly vulnerable to flooding by reducing the area available for water to infiltrate and increasing the speed of runoff.

The green infrastructure density of the area was classified into five classes 0–26.57(very high), 26.57–75.64(high), 75.64–145.15 (medium), 145.16–265.77(low), and 265.78–521.31(very low) which covers 60.42, 22.74, 11.28, 4.36, and 1.2 respectively, as shown in Fig. 5 (e) and Table 4. This implies that about 94.4 of the study are moderate to very highly vulnerable to flooding. This implies that the green infrastructure density of the area was very low to reduce flood vulnerability by increasing infiltration and reducing runoff.

Emergency centers (hospitals, schools, universities, and community centers) of the study area buffer were classified into five classes <500 m (very low), 500–1500 m (low), 1500–2500 m (medium), 2500–5000 m (high), and >5000 m((very high) which covers 15.86 %, 21.89 %, 22.73 %, 32.41 %, and 7.11 % respectively, as shown in Fig. 5 (f) and Table 4. This implies that about 62.25 % of the study area has moderate to very high vulnerability to flooding. This signifies that the availability of emergency centers such as hospitals, schools, and universities is less to assist the community during floods and serve as emergency response sites.

### 3.2 Analytical hierarchy processing (AHP)

The weight for all SET variables was taken into consideration as demonstrated in Table 4, reflecting the influence relative to each other. Total population (6.08 %), population density (6.77 %), percentage of children <5 years (6.64 %), percentage of population over 65 years(6.13 %), percentage of women(6.70 %), percentage of unemployed people (6.25 %). Additionally, slope variation (8.49 %), the proximity of the ecosystem to toxic release sites (7.04), a combination of shape index and average patch size (6.78 %), percentage of bare soil within the area (5.49 %), percentage of wetland within the area (5.32 %), productivity based on Normalized Difference in Vegetation Index (NDVI) (4.35 %). Furthermore, the percentage of the built area within the area (4.36 %), critical infrastructure in the area (4.38 %), road density (4.21 %), fractional impervious surface (3.49 %), green infrastructure density (3.46 %), and emergency centers (4.05 %).

**Fig. 8:**
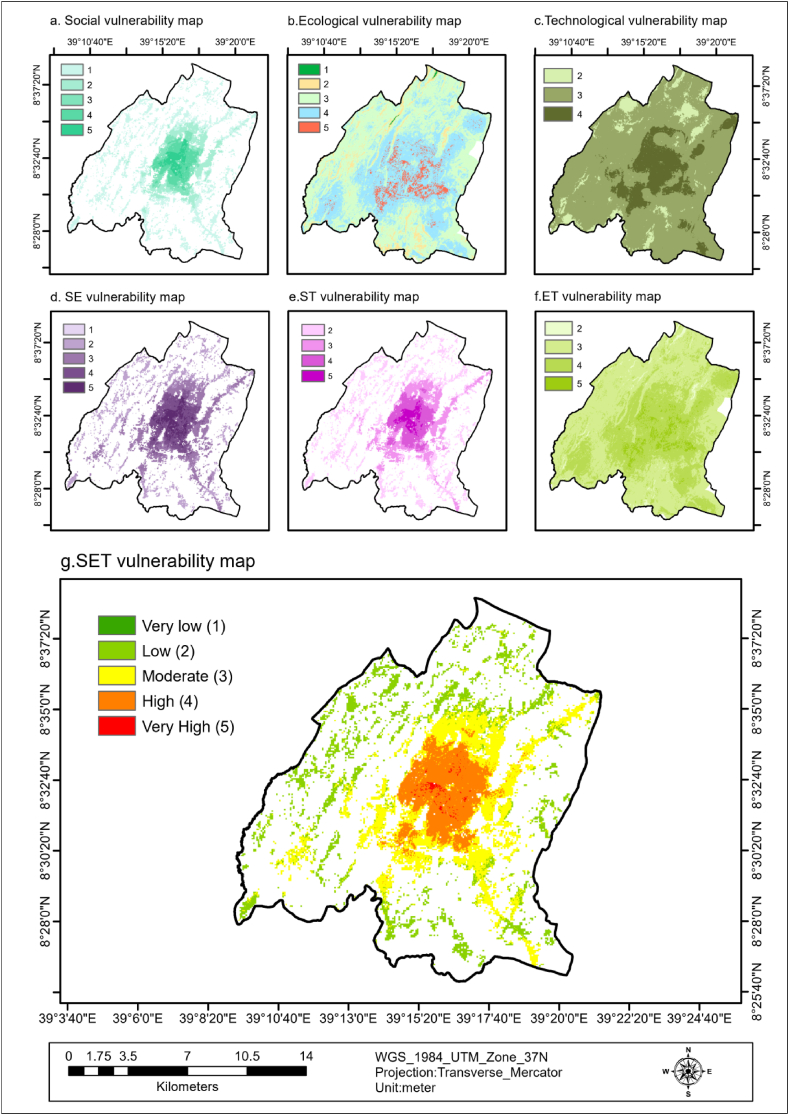
S, E, T, SE, ST, ET, SET flood vulnerability maps of the study area.

According to the social vulnerability map of the study area, approximately 28.32 % of the total area is moderate to very highly vulnerable to flooding, with 5.16 % (478.55 ha) as very high, 12.26 % (1136.35 ha) as high, 10.9 % (1010.55 ha) as moderate, 18.58 % (1722.36 ha) as low, and 53.11 % (4923.62 ha) as very low. The vulnerable area is located in the central part of the city, including Badhatuu, Odaa, Gurmuu, Dhadacha Araara, Abba Gadaa, Barreecha, Biqqa, Caffee, and Dagaaga, which are densely populated areas where a majority of the residents are women, children under five years, elderly over 65 years, and unemployed individuals. These findings were illustrated in Fig. 3(a–f) and Fig. 8 (a).

The ecological vulnerability map of the study area revealed that a significant percentage of the land is vulnerable to flooding. Specifically, 4.18 % is very highly vulnerable, 38.95 % is highly vulnerable, 50.9 % is moderately vulnerable, 5.85 % is lowly vulnerable, and 0.11 % is very lowly vulnerable. This indicates that an overwhelming majority of the total area (about 94.03 %) is susceptible to flooding, particularly in the densely urbanized regions characterized by flat terrain, proximity to toxic sites, smaller fragmented patches, bare soil areas, and less productive areas. These regions, which include Migiraa, Dagaaga, Dhadacha Araara, Gaara Luugo, and Irreechaa areas, are represented in Fig. 4(a–f) and Fig. 8 (b).

The technological vulnerability map of the study area revealed that 14.94% (4655.28 ha) is high, 77.17 % (24046.26 ha) is moderate and 7.88%(2456.69 ha) is low vulnerable to flooding. This implies that about 92.11 % of the area is technologically vulnerable to flooding. This area is characterized by most built-up areas, less availability of critical infrastructures, high road density, less impervious areas, and less green infrastructure density areas. These areas are all part of Dagaaga, Odaa, Badhatuu, Gurmuu, Abba Gadaa, Barreecha, Biqqa, and parts of Gaara.

Luugo, Migiraa, Irreechaa, and Gooro as shown in Fig. 5(a–f) and Fig. 8 (c).

According to the study's SET vulnerability map of the study area, flooding vulnerability levels varied across different neighborhoods. Specifically, 67.3 ha or 0.73 % of the area was identified as very highly vulnerable, while 2307.7 ha or 24.94 %, 3924.55 ha or 42.41 %, 2954.81 ha or 31.93 %, and 0.38 ha or 0.004 % were identified as high, moderate, low, and very low vulnerable, respectively. These findings indicate that approximately 68.08 % of the study area is at moderate to very high risk of flooding. In particular, local neighborhoods such as Dagaaga, Badhatuu, Odaa, Gurmuu, Abba Gadaa, Barreecha, Biqqa, and Dhadacha Araara were found to be among the most vulnerable (very high-to-high), while surrounding areas were deemed highly vulnerable to flooding. Factors contributing to this vulnerability include densely built environments, limited vegetation cover, high population density, proximity to toxic waste sites, unemployment, and fewer impervious surface areas, as illustrated in Fig. 8 (g).

#### 3.2.1 Spatial autocorrelation of SETS flood vulnerability maps

**Fig. 9:**
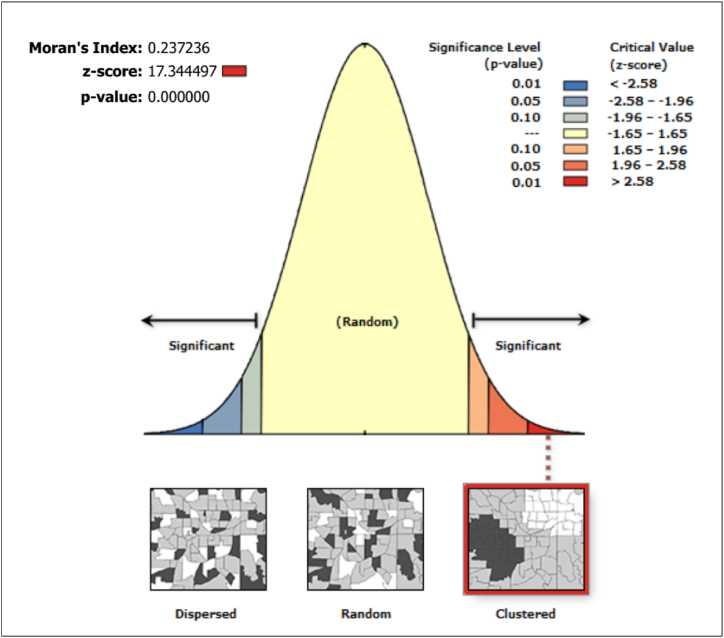
Spatial autocorrelation of SET flood vulnerability map.

According to the Global Moran's I summary, all SETS vulnerability maps exhibit clustering and positive autocorrelation (Table 8), as evidenced by the positive Moran's I index values. In particular, for the SET vulnerability map displayed in Fig. 9, Moran's I Index was 0.237236, indicating a positive spatial autocorrelation in the map. This implies that similar values tended to be clustered together in space rather than being randomly distributed. The Expected Index was close to zero (−0.000181), suggesting the absence of any spatial pattern if the data were randomly distributed. The data variance (0.000187) describes the degree of variation present, whereas the Z score (17.344497) indicates a significant difference between the observed and expected indices. The p-value (0.000000) being less than the significance level of 0.05 provides strong evidence to reject the null hypothesis that the spatial pattern is random. Consequently, the Global Moran's I Summary result suggests a clustered spatial pattern for SET vulnerability maps.

**Table 8:** Global Moran's I Summary for S, E, T, SE, ST, ET, and SET flood vulnerability maps.

Global Moran's I Summary	S	E	T	SE	ST	ET	SET
Moran's Index	1.136199	0.283748	0.205036	0.445320	0.671565	0.147702	0.237236
Expected Index:	−0.000818	−0.000051	−0.000356	−0.000151	−0.000586	−0.000071	−0.000181
Variance:	0.001418	0.000036	0.000229	0.000151	0.000829	0.000051	0.000187
z-score	30.196861	47.099950	13.580173	36.266498	23.347086	20.648194	17.344497
p-value	0.000000	0.000000	0.000000	0.000000	0.000000	0.000000	0.000000
Spatial pattern	Clustered	Clustered	Clustered	Clustered	Clustered	Clustered	Clustered

A clustered spatial pattern in Global Moran I for flood vulnerability indicates that areas with high flood vulnerability tend to be clustered together, and this has important implications for the allocation of resources and mitigation strategies. Understanding the clustering of different areas can aid in prioritizing interventions and allocating resources effectively to mitigate flood vulnerability. This information is depicted in Fig. 8(a–g), which presents separate, combined, and overall SET flood vulnerability maps, allowing for targeted actions with maximum impact. It can also help identify areas where measures such as building codes or land use regulations can be implemented to reduce vulnerability cost-effectively. Overall, understanding the clustered spatial pattern of flood vulnerability can provide valuable insight for decision-makers and planners seeking to reduce the impact of floods on communities and infrastructure.

## 4 Discussions

Analyzing urban flooding complexities through the SETS approach is essential as it examines interdependencies between social, ecological, and technological systems that escalate flood vulnerability of residents in flood-prone areas due to changes in land use, hydrology, population, economic growth, and climate [15,20,69]. Due to this, It is imperative to give due consideration to the SETS framework as it facilitates an analysis of floods on both an individual and interconnected basis. This is especially the case in developing countries, where limited infrastructure and resources exacerbate the severity of flooding impacts [29,99,100]. The findings of the study revealed that locally known neighborhoods such as Badhatuu, Odaa, Gurmuu, Dhadacha Araara, Abba Gadaa, Barreecha, Biqqa, Caffee, and Dagaaga face the risk of negative impacts from flooding events. These areas have high rates of unemployment, significant elderly and child populations under five years of age, and predominantly women residents. The findings of the current study are consistent with studies conducted by Refs. [20,69,101] as flood exposure and social vulnerability are interconnected, and socially vulnerable populations, including children, the elderly, the disabled, the poor, and minorities, are more likely to experience injury and death from flooding relative to the others.

The vulnerability of floods was also heightened in locations, where depressed and flat areas are prevalent, proximity to toxic release sites from industries, smaller and fragmented patches, and bare soil areas. Those areas were found within the study area locally known as Migiraa, Dagaaga, Dhadacha Araara, Gaara Luugo, and Irreechaa areas. This study supports the findings of prior research indicating that flatter topographies are prone to flooding and have lower slope values [73]. Vegetation cover is seen as an effective means of mitigating flood vulnerability by reducing surface runoff and absorbing water, which is in agreement with studies conducted by Cian, Marconcini [74], de la Iglesia Martinez and Labib [76], and Zhang, Chang [75]. Moreover, proximity to toxic industries, where the potential for exposure to hazardous waste poses risks to surrounding communities as conducted by Chang, Pallathadka [15], Jongman, Winsemius [78], and Talbot, Bennett [7]. Similarly, fragmented and smaller patches of ecosystems are less effective at providing flood mitigation services, as demonstrated in studies by Bousquin and Hychka [80]; and Rehman, Hasan [79]. In addition, bare soil areas pose a greater risk in terms of their low infiltration capacity and high erodibility in the event of floods, as posited by Ref. [15].

Areas characterized by most built-up areas, less availability of critical infrastructures, high road density, less impervious areas, and less green infrastructure density areas which are located in the neighborhoods of Dagaaga, Odaa, Badhatuu, Gurmuu, Abba Gadaa, Barreecha, Biqqa and parts of Gaara Luugo, Migiraa, Irreechaa, and Gooro are vulnerable to flooding. This finding is consistent with previous studies done in different urban areas [39]. The inability of the built-up areas to allow water to percolate through the subsurface makes them more vulnerable in the study area, which is consistent with the study conducted by Deepak, Rajan [82].

The presence of high fractions of impervious surfaces and dense road networks also exacerbate the risk of floods by impeding water infiltration and accelerating surface runoff [77,83]. Consequently, these factors contribute significantly to flood vulnerability within the study area. The aforementioned studies have established this connection between the lack of subsurface permeability and the increased susceptibility of built-up areas to flooding, as well as the role played by impervious surfaces and road density in exacerbating flood risks.

The study reveals that the area characterized by a lower density of green infrastructure also faces increased susceptibility to flooding. This poses a hindrance to infiltration processes and exacerbates runoff. These findings further support a previous study, conducted by Khodadad, Aguilar-Barajas [86] and Pallathadka, Sauer [85], which highlights the crucial role of green infrastructure in mitigating an area's vulnerability to flooding through its ability to enhance infiltration and reduce runoff. The consistency between these studies reinforces the significance of incorporating green infrastructure as a means to alleviate flood-related risks in urban environments. This correlation underscores the importance of pursuing strategies that prioritize the integration of green infrastructure to enhance the resilience of areas prone to flooding.

According to the findings of the present study, certain areas close to critical infrastructure such as toxic industries, landfill sites, abattoirs, and gas terminals face a significant vulnerability to flooding, thus posing a threat to the local community. This finding aligns with previous studies conducted by Murdock, De Bruijn [88], Qiang [89], Lin, Wu [94], who highlighted the potential flood vulnerability associated with proximity to such infrastructure. Conversely, this study found that areas near emergency centers, including hospitals, schools, and universities, play a crucial role during floods by acting as essential assets for support and serving as emergency response sites. This finding is consistent with the previous research carried out by Atanga and Tankpa [90], Cutter, Boruff [91], further emphasizing the importance of these locations in flood response and underscoring their significance in safeguarding affected communities.

## 5 Conclusion

This research highlights the significance of applying the SETS framework to characterize the flood vulnerability of urban areas such as Adama City. The findings indicated that vulnerability to flooding across the SETS domains follows a clustered distribution pattern. Moreover, the study identified spatial correlations between S-E, S-T, and E-T vulnerabilities, indicating opportunities to enhance flood mitigation measures in multiple domains simultaneously. This research can provide insights into developing more effective flood vulnerability reduction strategies in Adama City and other urban areas facing similar challenges.

The study explored the implications of sustainable urban development, with specific emphasis on exposure, sensitivity, and adaptability to flood vulnerability. The findings of the research have significant implications for cities undertaking sustainable urban development. Based on the SET vulnerability map developed for the study area, it was observed that the levels of vulnerability to flooding differed among various neighborhoods. The results highlighted that approximately 68.08 % of the study area was exposed to moderate to very high flood vulnerability, necessitating immediate attention and mitigation efforts. By utilizing the SETS framework, a better understanding of the interconnectivity between environment, infrastructure, and equitable distribution of hazards within society was gained, which is critical in achieving sustainable urban development. The relevance to other developing cities is that by applying the SETS framework initially tested in Adama City, municipalities can benefit from these findings and evaluate their flood vulnerability status. As flooding is spatially confined to specific areas, municipal administrators and practitioners can effectively target vulnerable neighborhoods to adapt to flooding. Ultimately, this study provides insights into an improved understanding of the complex nature of sustainable urban development, particularly regarding natural hazard management. Additionally, this study also helps to ensure that the SETS frameworks to tailored to the specific needs and concerns of each community, reflecting their unique circumstances and priorities.

## Data availability statement

No additional information is available for this paper.

## CRediT authorship contribution statement

**Bikila Merga Leta:** Conceptualization, Data curation, Formal analysis, Investigation, Methodology, Software, Validation, Writing – original draftWriting – original draft, Writing – review & editingWriting – review & editing. **Dagnachew Adugna:** Supervision, Validation, Writing – review & editingWriting – review & editing.

## Declaration of competing interest

The authors declare that they have no known competing financial interests or personal relationships that could have appeared to influence the work reported in this paper.
